# Amyloid-β Processing in Aged S100B Transgenic Mice Is Sex Dependent

**DOI:** 10.3390/ijms221910823

**Published:** 2021-10-06

**Authors:** Krista Minéia Wartchow, Leticia Rodrigues, Izabela Swierzy, Michael Buchfelder, Diogo Onofre de Souza, Carlos-Alberto Gonçalves, Andrea Kleindienst

**Affiliations:** 1 Department of Biochemistry, Federal University of Rio Grande do Sul, Porto Alegre 90035-003, Brazil; kristawartchow@hotmail.com (K.M.W.); letigues@gmail.com (L.R.); diogo@ufrgs.br (D.O.d.S.); casg@ufrgs.br (C.-A.G.); 2 Department of Neurosurgery, Friedrich-Alexander University, 91054 Erlangen, Germany; izabela.swierzy@uk-erlangen.de (I.S.); Michael.Buchfelder@uk-erlangen.de (M.B.)

**Keywords:** Alzheimer’s disease, amyloid-β processing, S100B, transgenic mice, neurodegeneration

## Abstract

(1) Background: Calcium-binding protein S100B is involved in neuroregeneration but has also been associated with neurodegeneration. These contrasting effects may result from concentration or duration of exposure. We investigated the effect of long-term increased S100B levels on amyloid-β processing in one-year-old transgenic (tg) mice with 12 copies of the murine S100B gene with specific consideration of sex and specific brain regions. (2) Methods: S100B and amyloid-β 42 (Aβ42) were quantified in serum, cerebrospinal fluid (CSF), adipose tissue, and different brain regions by ELISA in wild-type (wt) and S100Btg mice (each *n* = 7 per group). Thioflavin T (ThT) and Aβ immunostaining were performed for visualization of Aβ deposition. (3) Results: S100B in serum, CSF, and brain was significantly increased in S100Btg mice of both sexes. Aβ42 was significantly increased in the hippocampus of male S100Btg mice (*p* = 0.0075), and the frontal cortex of female S100Btg mice (*p* = 0.0262). ThT and Aβ immunostaining demonstrated Aβ deposition in different brain regions in S100Btg mice of both sexes and female wt. (4) Conclusion: Our data validate this experimental model for studying the role of S100B in neurodegeneration and indicate that Aβ processing is sex-dependent and brain region-specific, which deserves further investigation of signaling pathways and behavioral responses.

## 1. Introduction

Calcium-binding protein S100B is most abundant in brain tissue, mainly expressed and secreted by astrocytes [1], and exerts dose-dependent either neurotrophic or neurotoxic effects (for review, see [2]). Extracellular S100B levels have been proposed as a marker of brain injury, particularly in blood [3] and cerebrospinal fluid (CSF) [3,4]. One potential contributor to increased S100B serum levels is the adipose tissue since adipocytes express S100B [5].

The S100B gene is localized on chromosome 21 [6], and hence, S100B has been associated with Down syndrome and Alzheimer’s disease (AD) [7]. S100B expression correlates with the density of neuritic plaques [8], and dystrophic neurites overexpressing β-amyloid precursor protein [9]. Whether the increase in S100B in neurodegenerative diseases is part of a compensatory response, contributes to the pathology, or results from dysregulated feedback loops has not been clarified yet. On the other hand, the therapeutic short-term increase in S100B levels promoted hippocampal neurogenesis in rodents, even following experimental brain injury [10,11].

Transgenic mice overexpressing S100B were developed in the early 1990s with variable numbers of S100B gene copies [12,13], which provide an experimental tool for better understanding the contribution of S100B in neuroregeneration or -degeneration. In vivo studies in S100Btg mice demonstrated electrophysiological and behavioral alterations, such as depression of post-tetanic excitatory postsynaptic potentials, impairment of spatial learning, abnormal exploratory behavior, and hyperactivity in the open field [14,15,16]. Hippocampal structural changes included astrogliosis, i.e., excess accumulation of glial fibrillary acidic protein (GFAP) beyond S100B, and axonal sprouting [12]. Age-dependent, S100Btg mice demonstrated dendritic cytoskeletal changes [17].

A potential mechanistic relationship between S100B and amyloid-β plaques was investigated by crossing S100Btg mice and Tg2576 mice, which overexpress amyloid-β precursor protein (AβPP) [18]. The authors demonstrated that S100B overexpression accelerates AD-like pathology. Griffin et al. proposed that interleukin-1β induces astrocyte activation and astrocytic overexpression of S100B, which, in turn, results in neuronal AβPP production thereby promoting the inflammatory cycle underlying AD [9]. In support of this hypothesis, we demonstrated interleukin-1β to induce S100B secretion in glial cultures and hippocampal slices [19]. In vivo, S100B extracellular levels have not been evaluated in S100Btg mice yet, at least to the best of our knowledge, neither extracellular amyloid-β peptide levels nor amyloid-β deposition. Due to the escalating prevalence of AD in humans, current research on the role of S100B in AD concurs at many fronts including neuroinflammation [20], brain energy metabolism [21], AβPP processing, and turn-over [22] as well as at the neuromarker level [23]. 

Here, we investigated the implication of long-term 10-fold increased nanomolar S100B concentrations in transgenic mice with 12 copies of the murine S100B gene on neurodegeneration or AD, and whether increased amyloid-β peptide levels depend on the sex or specific brain regions. Specifically, we quantified S100B and amyloid-β 42 (Aβ_42_) peptide levels in the blood serum, CSF, adipose tissue, and different brain regions of one-year-old S100Btg female and male mice. Additionally, we assessed brain amyloid-β deposition qualitatively and quantitatively, and to address specific aspects of aging, we compared the latter results with three-month-old mice. Our data demonstrate specific changes in the brain tissue and CSF following long-term S100B exposure in aged S100Btg mice and indicate that subsequent S100B level and amyloid-β processing are sex dependent.

## 2. Results

To evaluate the participation of S100B in Aβ pathology in aged S100Btg mice, we utilized two strategies. First, we quantified S100B and Aβ_42_ peptide levels in the blood serum, CSF, adipose tissue, and different brain regions by ELISA analysis. Second, we assessed the spatial Aβ antibody immunoreactivity (conventional Aβ “burden”) qualitatively and deposition with Thioflavin T fluorescence, which has an affinity for amyloid-β fibrils, quantitatively.

### 2.1. Central and Peripheral S100B Levels in S100Btg Mice

**Figure 1:**
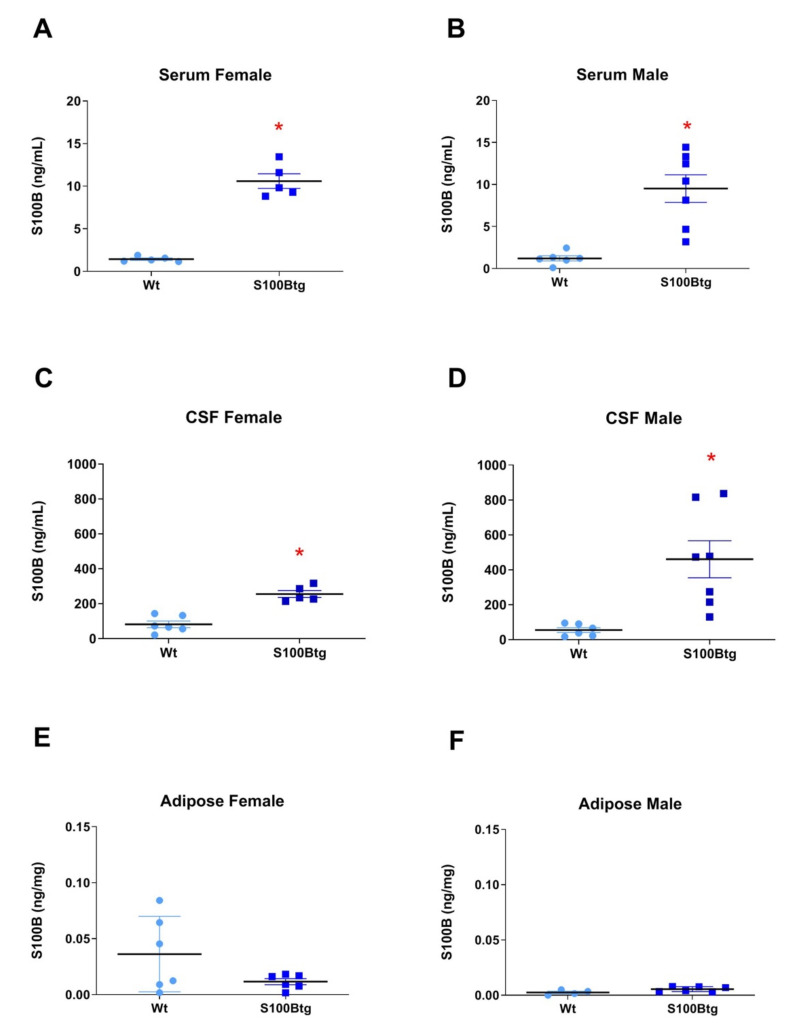
S100B level in blood serum, cerebrospinal fluid, and adipose tissue in one-year-old S100Btg and wt mice: in (**A**,**B**) serum concentration of S100B in females and males; in (**C**,**D**), cerebrospinal fluid (CSF) concentration of S100B in females and males; in (**E**,**F**), adipose tissue content of S100B in females and males; measurements performed by ELISA. Data are expressed as means ± SE (7 mice/group). * indicates a statistically significant difference (Student’s *t*-test, assuming *p* < 0.05).

In *serum*, the S100B concentration (S100B_serum_) was increased in female (Figure 1A, *p* < 0.0001) and male (Figure 1B, *p* = 0.0008) S100Btg mice, as compared with wt mice. In the *CSF*, the S100B concentration (S100B_CSF_) was increased in female (Figure 1C, *p* = 0.0001) and male (Figure 1D, *p* = 0.005) S100Btg mice, as compared with wt mice. In the *adipose tissue*, a potential major extracerebral source of S100B [24], the S100B content was neither increased in female S100Btg mice (Figure 1E) nor in male S100Btg mice (Figure 1F), as compared with wt mice.

**Figure 2:**
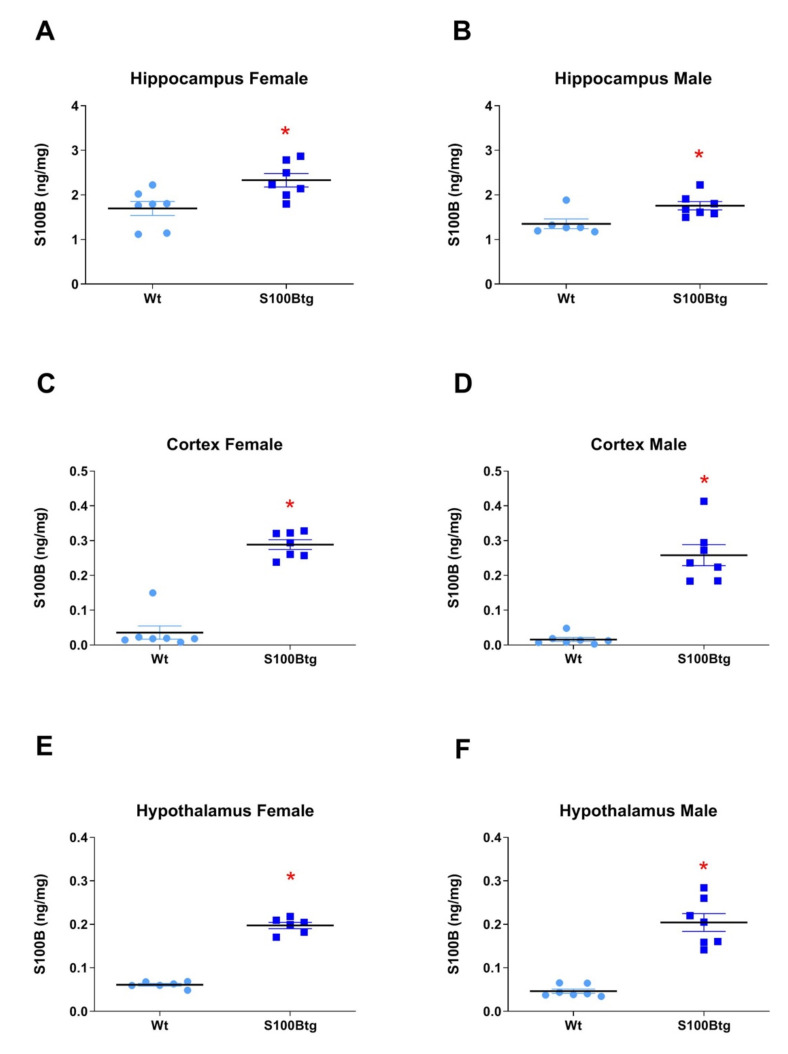
S100B brain content in one-year-old S100Btg and wt mice**.** S100B content was quantified in (**A**,**B**) hippocampus, (**C**,**D**) frontal cortex, and (**E**,**F**) hypothalamus; measurements were performed by ELISA. Data are expressed as means ± SE (7 mice/group). * indicates a statistically significant difference (Student’s *t*-test, assuming *p* < 0.05).

The *S100B brain content* was measured in three different regions: hippocampus, frontal cortex, and hypothalamus. In all three brain regions, the S100B content was increased in S100Btg mice, i.e., in female (hippocampus: Figure 2A, *p* = 0.0134; frontal cortex: Figure 2C, *p* < 0.0001; hypothalamus: Figure 2E, *p* < 0.0001) and in male (hippocampus: Figure 2B, *p* = 0.0157; frontal cortex: Figure 2D, *p* < 0.0001; hypothalamus: Figure 2F, *p* < 0.0001) S100Btg mice, as compared with wt mice.

### 2.2. Amyloid β_42_ Levels and Aβ Immunoreactivity in S100Btg Mice

**Figure 3:**
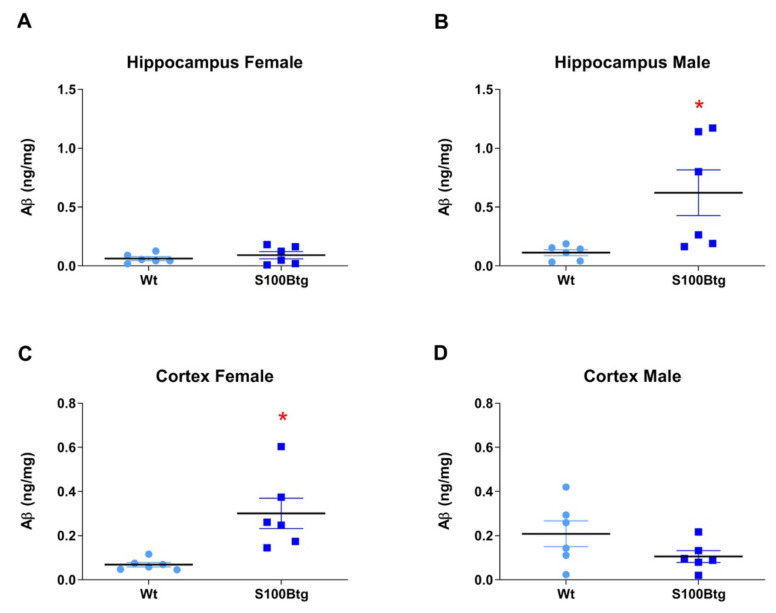
Brain levels of amyloid β-peptide 1-42 (Aβ_42_) in one-year-old S100Btg and wt mice. Aβ_42_ levels were quantified in (**A**,**B**) hippocampus and (**C**,**D**) cortex; measurements were performed by ELISA. Data are expressed as means ± SE (7 mice/group). * indicates a statistically significant difference (Student’s *t*-test, assuming *p* < 0.05).

**Figure 4:**
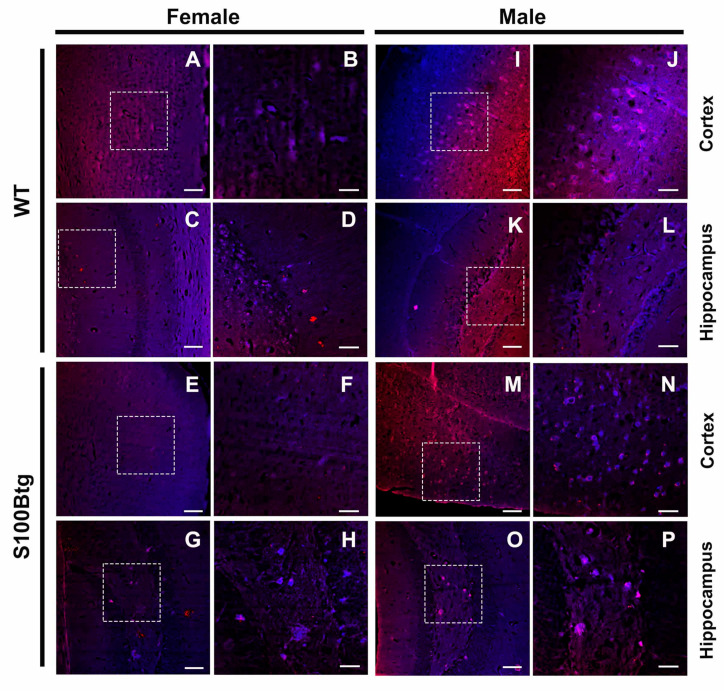
Brain amyloid-β deposition in one-year-old S100Btg and wt mice. In (**A**–**H**), Aβ_42_ immunofluorescent images of female wt and S100Btg mice, magnified by 10× and 40×, respectively. (**B**,**D**,**F**,**H**) are higher magnifications corresponding to the squares in (**A**,**C**,**E**,**G**), respectively. In (**I**–**P**), fluorescent images of male wt and S100Btg mice, magnified by 10× and 40×, respectively. (**J**,**L**,**N**,**P**) are higher magnifications corresponding to the squares in (**I**,**K**,**M**,**O**), respectively. Scale bars = 150 µm (left panels) and 50 µm (right panels).

The *Aβ_42_ brain content* was evaluated in two different regions: hippocampus and frontal cortex. In the hippocampus, the Aβ_42_ ELISA content was increased in male S100Btg mice (Figure 3B, *p* = 0.0262; note the specific immunostaining in the hilus region of the hippocampus Figure 4G,H) but not in female S100Btg mice (Figure 3A and Figure 4O,P), as compared with wt mice (Figure 4C,D,K,L). In the frontal cortex, the Aβ_42_ ELISA content was increased in female S100Btg mice (Figure 3C, *p* = 0.0075; Figure 4E,F) but not in male S100Btg mice (Figure 3D and Figure 4M,N), as compared with wt mice (Figure 4A,B,I,J).

### 2.3. Correlation of S100B and Amyloid β_42_ in S100Btg Mice 

**Figure 5:**
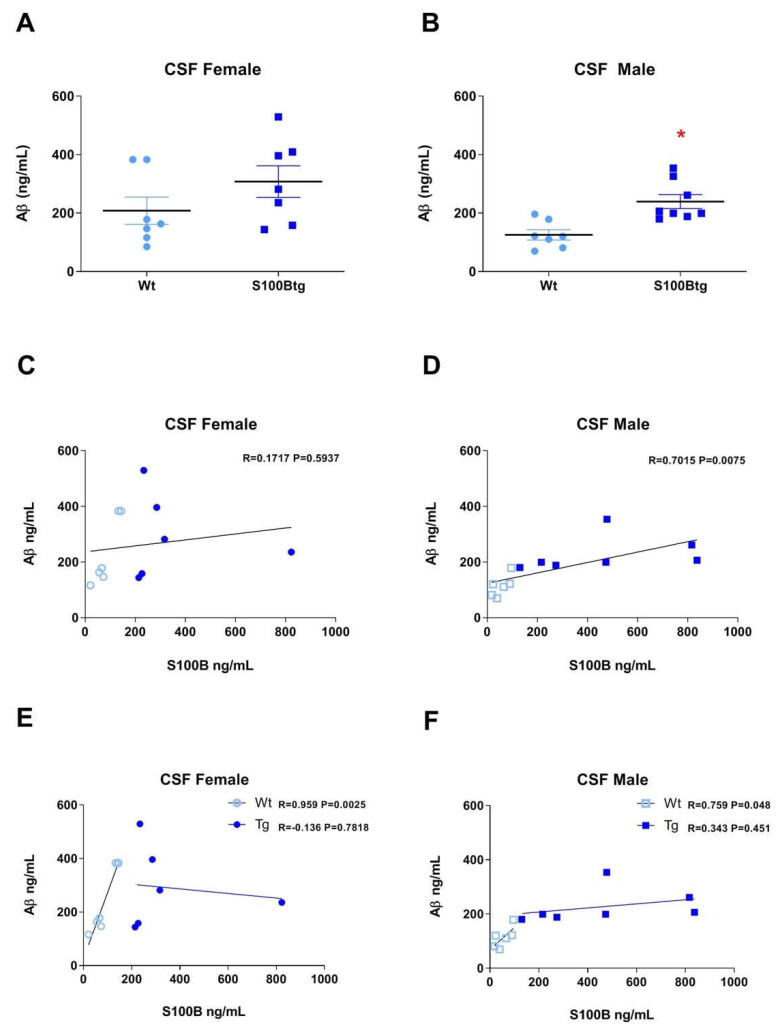
CSF levels of amyloid β-peptide 1-42 (Aβ_42_) and correlation of S100B and amyloid β_42_ in one-year-old S100Btg and wt mice. Aβ_42_ levels were quantified in (**A**,**B**) the cerebrospinal fluid (CSF), * indicates a statistically significant difference (Student’s *t*-test, assuming *p* < 0.05); (**C**–**F**) we determined correlations between S100B_CSF_ and Aβ_42 CSF_ in wt and S100Btg mice, as analyzed by the Pearson correlation coefficient, assuming *p* < 0.05.

In the *CSF*, the Aβ_42_ concentration (Aβ_42 CSF_) was slightly increased in female S100Btg mice (Figure 5A, *p* = 0.1877) and significantly increased in male S100Btg mice (Figure 5B, *p* = 0.0024), as compared with wt mice. 

S100B_CSF_ was not significantly correlated with Aβ_42 CSF_ in female mice (Figure 5C, *r* = 0.5937, *p* = 0.1717) but correlated in male mice (Figure 5D, *r* = 0.7015, *p* = 0.0075). However, with regard to a group-specific correlation, Aβ_42 CSF_ was correlated with S100B_CSF_ (female Figure 5E, *p* = 0.003; *r* = 0.959; male Figure 5F, *p* = 0.048; *r* = 0.759) in wt but not in S100Btg mice (female Figure 5E, *p* = 0.798; *r* = −0.136; male Figure 5F, *p* = 0.451; *r* = 0.343).

In the *hippocampus* of wt mice, Aβ_42_ was not correlated with S100B (female *p* = 0.823; *r* = 0.119; male, *p* = 0.205; *r* = −0.603). In female S100Btg mice, the correlation was negative (*p* = 0.001; *r* = −0.972). In male S100Btg mice, Aβ_42_ was not correlated with S100B (*p* = 0.381; *r* = 0.395).

In the *frontal cortex* of wt mice, Aβ_42_ was not correlated with S100B (female *p* = 0.696; *r* = 0.101; male *p* = 0.291; *r* = −0.519). In female S100Btg mice, there was no correlation (*p* = 0.370; *r* = 0.751). In male S100Btg mice, Aβ_42_ was correlated with S100B (Figure 5F, *p* = 0.009; *r* = 0.920).

### 2.4. Amyloid-β Aggregation as Assessed by Thioflavin T Fluorescence 

**Figure 6:**
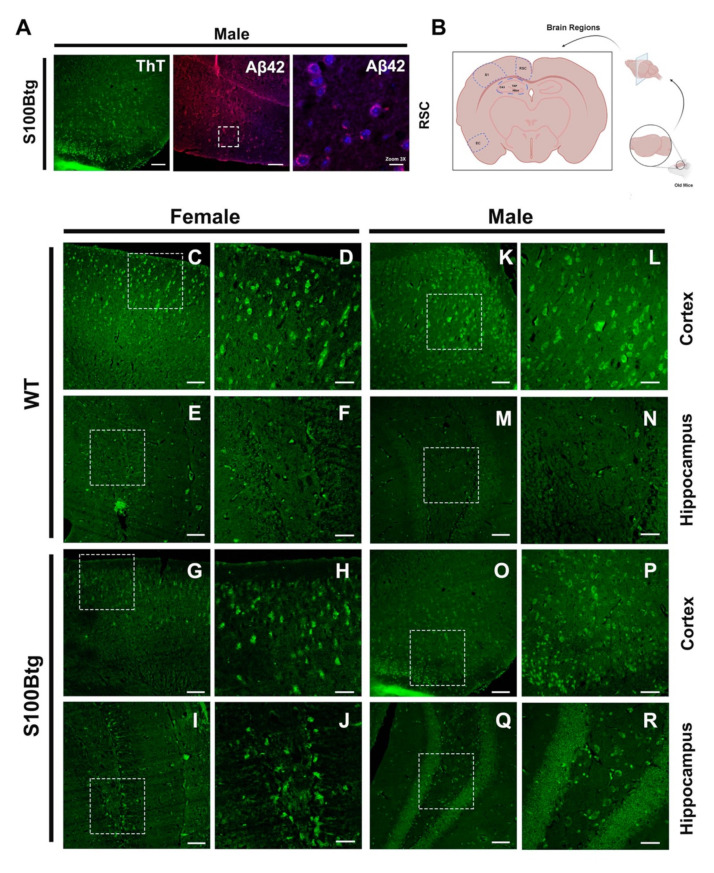
Brain amyloid-β deposition in S100Btg mice. In (**A**), pattern of labeling comparing ThT and amyloid-β immunofluorescence. (**B**), brain regions, the dorsal hippocampus and cortex, of S100B transgenic and wild-type mice investigated by Thio-flavin T (ThT) affinity. In (**C**–**R**), Tht fluorescent images of male and female brains of wt and S100Btg mice, magnified by 10× and 40×, respectively. Scale bars = 150 µm (left panels) and 50 µm (right panels).

To validate increased Aβ_42_ brain and CSF levels in S00Btg mice, we evaluated potential amyloid-β plaques or similar aggregation deposits with Aβ_42_ immunostaining qualitatively, and with ThT staining quantitatively (Figure 6A).

Since the Aβ_42_ staining suggested an amyloid aggregation in the hilus of the hippocampus, we quantified the ThT fluorescence of different hippocampal pathways, i.e., the temporoammonic pathway (*TAP*, monosynaptic projection from entorhinal cortex layer 3 to the CA1 region; involved in spatial memory learning), the *CA3* region (termination of entorhinal cortex layer 2; novelty detection and short-term memory), and the hilar mossy cells of the dentate gyrus (*hilus*; dynamic regulation of pattern separation). The evaluated areas are depicted in Figure 6B. However, there were no regional differences of ThT fluorescence in the hippocampal pathways evident, neither in wt nor in S100Btg mice (Table 1). Following qualitative Aβ_42_ immunohistochemistry, ThT fluorescence was significantly increased in male S100Btg mice (female Figure 6I,J, non-significant; male Figure 6Q,R, *p* < 0.001), as compared with their wt counterparts (Figure 6M,N). Concerning gender differences in wt mice, female one-year-old mice demonstrated a significantly higher ThT fluorescence than male ones (Figure 6E,F,M,N, *p* < 0.001). Within the cortex, the ThT fluorescence was not consistent different between S100Btg and wt mice (Figure 6C,D,G,H,K,L,O,P). Quantitative analysis of ThT fluorescence and Aβ_42_ levels measured by ELISA demonstrated some discrepancies, but in the latter, we were looking for soluble peptide content, while ThT fluorescence demonstrates fibrillar or insoluble amyloid aggerates.

As even one-year-old wt mice demonstrated reasonable yet intriguing amyloid aggregation, we pursued another set of ThT staining and quantification in 3-month-old mice. In wt mice, the ThT fluorescence was significantly increased at one year of age, and in male wt mice, already at three months of age (Table 1). Concerning *age-dependent differences,* the relative ThT fluorescence at an age of three months did not differ among wt and S100Btg mice of any sex. However, with increasing age, the ThT fluorescence was significantly pronounced in S100Btg mice of both sexes and female wt mice (each *p* < 0.001).

## 3. Discussion

Here, we presented data of S100B long-term exposure on neurodegeneration in one-year-old transgenic mice in terms of amyloid-β peptide levels or amyloid-β deposition with specific consideration of sex-dependent effects. We reported a significant region-specific amyloid-β accumulation in S100Btg mice, i.e., in the frontal cortex in female S100Btg mice and the hippocampus in male S100Btg mice. ThT staining demonstrated plaque-like amyloid-β deposits, not as much evident at three months of age. The correlation between S100B and Aβ_42_ levels was different when comparing S100Btg and wt mice. 

### 3.1. S100B Serum and CSF Levels in One-Year-Old S100Btg Mice

Given that S100B participates in brain development [25,26], S100B levels are highest in neonatal animals and humans, thereafter dropping with increasing ages. However, in a variety of acute and chronic brain pathologies, including traumatic brain injury, ischemic insult, and neurodegenerative diseases, S100B_serum_ levels have been found to increase again (review [27]). Early on, following brain insults, S100B induces upregulation of the anti-apoptotic factor Bcl-2, thereby promoting neuronal repair [28], while later, deleterious effects are mediated through overexpression of AβPP protein with subsequent formation of dystrophic neurites and deposition of amyloid-β plaques, ultimately resulting in neurodegenerative diseases such as AD [29]. Chronically elevated S100B_serum_ levels are found in Down syndrome, i.e., trisomy of the S100B encoding chromosome 21 [30]. 

In this study, utilizing S100Btg mice with the insertion of 12 copies of the murine S100B gene, S100B_serum_ levels were increased 10-fold. Another S100Btg mouse line with insertion of 70 copies of the human S100B gene expressed fivefold increased S100B levels and revealed an accelerated hippocampal development, followed by enhanced aging [17,31]. 

It is important to emphasize that S100B_serum_ levels do not necessarily reflect S100B_CSF_ levels, since either extracerebral sources may contribute to serum levels or the passage of S100B from CSF to serum may vary. The most likely source of extracerebral S100B is the adipose tissue since adipocytes secrete S100B [24,28,29]. In this study, the S100B content in the adipose tissue of S100Btg mice was not different from that of wt mice, at least at one year of age. S100B mRNA levels do not always correspond to the respective protein levels, suggesting a complex gene regulation and cell-type dependence [5,32,33]. At this time, it remains unclear why S100Btg mice express relatively low levels of protein in adipose tissue. Therefore, this S100Btg mouse model is distinguished for studying central nervous system pathologies. Another reason why S100B_serum_ levels may not correlate with S100B_CSF_ is alterations in the blood–brain or blood–CSF barrier. We previously reported acute conditions of brain injury associated with S100B_CSF_ changes that are not accompanied by S100B_serum_ variations [34,35]. 

In this study, S100B_CSF_ levels were increased in S100Btg mice 10-fold, compared with S100B_serum_ levels, reflecting an unaltered passage from the brain extracellular fluid to CSF and further to serum. To the best of our knowledge, this is the first time that extracellular S100B levels have been measured in S100Btg mice. The increased extracellular S100B levels support the hypothesis that S100B, released by astrocytes, could affect neurons in the neighborhood. Moreover, the low S100B levels in adipose tissue in S100Btg mice favor this model for central nervous system diseases and subsequent alterations of the brain–CSF–blood transport since adipocytes as extracerebral contributors can be excluded.

### 3.2. S100B and Aβ_42_ Brain Content in One-Year-Old S100Btg Mice

Quantifying cellular and extracellular brain tissue content in S100Btg mice, we found increased levels in all the three examined brain regions, i.e., hippocampus, frontal cerebral cortex, and hypothalamus. In the hippocampus, S100B levels were higher than in the other two regions, even in wt mice. However, since S100B in brain tissue was quantified by ELISA, we were not able to verify any cellular preference of S100B expression to hippocampal stellate astrocytes and other glial cells as reported originally [12]. 

Chronic neuroinflammation results in neuronal AβPP expression, increased Aβ_42_, and ultimately amyloid-β plaques formation seen in AD, and is most likely mediated through the pluripotent cytokine interleukin-1 (IL-1) [36]. Although IL-1 is known to also increase S100B release, other authors suspect S100B to be the causal factor [18]. Increased S100B_CSF_ occurs during the mild-cognitive impairment phase of AD [37], suggesting that the elevation of extracellular S100B may alternatively be an endogenous neuroprotective attempt. Accordingly, in the streptozotocin-model of sporadic AD, we observed an increase in S100B_CSF_ in the first week, and four weeks later, a decrease [20]. 

S100B_serum_ has positively and negatively been associated with cognitive function in older patients [38,39], and a recent meta-analysis demonstrated increased S100B_serum_ but not S100B_CSF_ in AD patients [23]. This difference between S100B_serum_ and S100B_CSF_ levels may reflect an altered brain–CSF–blood barrier due to neuroinflammation in AD patients. As mentioned above, in our S100Btg mice, the increase of S100B_serum_, S100B_CSF_, and brain levels was concordantly suggesting an unaltered brain–CSF–blood barrier. 

### 3.3. Correlation of S100B and Aβ_42_ Levels in One-Year-Old S100Btg Mice

We could demonstrate a positive correlation of S100B_CSF_ and Aβ_42 CSF_ only within the physiological range in wt mice but not at more elevated levels in S100Btg mice, and even more importantly, neither in wt nor in S100Btg mice in the brain tissue. Nevertheless, both S100B_CSF_ and Aβ_42 CSF_ levels were increased in S100Btg, as compared with wt mice. We cannot affirm definitively a clear causal relationship between S100B and Aβ_42_, although recent evidence has shown an interaction between S100B and amyloid-β processing with anti-aggregation properties [22,40,41]. The loss of correlation between S100B and Aβ_42_ levels in S100Btg mice may be due to the chronically elevated S100B levels affecting neuron-glial communication observed at physiological levels.

Due to the *sex dependence* of AD (review [42]), attention has to be paid to sex bias analyzing experimental data. Approximately two-thirds of AD cases occur in women, and in the US, AD is the 5th cause of death in women and t8th in men. In Tg2475 mice overexpressing the human AβPP, the quantitative analysis of silver AD staining in the hippocampus and cortex reveals slightly elevated Aβ_42_ levels and amyloid-β plaques at 12 months in females, reaching statistical significance at 15 months [43]. While pioneer work with S100Btg mice demonstrated behavioral sex-dependent alterations [14], there is a gross lack of continuing research. In our study, the Aβ_42_ brain content in one-year-old S100Btg mice is modulated by sex and regions. In male S100Btg mice, Aβ_42_ levels were increased in the hippocampus, while in female S100Btg mice, Aβ_42_ levels were increased in the frontal cortex. It is possible that female mice of this age are still protected against amyloid-β toxicity by estrogen [44] and further experiments with older S100Btg mice have to be amended.

### 3.4. Indication of Amyloid-β Aggregations in S100Btg Mice

Histopathological indicators of AD are extracellular amyloid-β plaques (resulting from the deposition of Aβ peptides 1–40 and 1–42 of the neuronal AβPP protein) and intraneuronal fibrillary tangles (resulting from an abnormal microtubule organization, due to tau hyperphosphorylation). While human S100B and AβPP are both encoded in the same region of chromosome 21, and in postmortem brains of AD patients, S100B directly correlates with the number of neuritic plaques [8], no proof exists yet for a direct causal relationship. Even though AβPP is present in rodents, no spontaneous murine AD has been reported. However, the insertion of the human AβPP gene in rodents is a widely used model for AD. Tg2475 mice, for example, overexpress a mutant form of AβPP (Swedish mutation), resulting in elevated Aβ levels and, subsequently, amyloid-β plaques [45]. Likewise, the induction of sporadic AD by intracerebroventricular streptozotocin accelerates amyloid-β plaques in Tg2475 mice [46], results in tau hyperphosphorylation (found in neurofibrillary tangles) and amyloid-β deposition around capillaries (review [47]). 

Following Aβ_42_ immunohistochemistry, we quantified the density of amyloid-β deposits with ThT staining. Since the dorsal hippocampus is especially susceptible to neurodegeneration [48], and Aβ_42_ staining in our series suggested an amyloid aggregation in the hilus of the hippocampus, we evaluated the ThT staining of different dorsal hippocampal pathways but could not verify any regional differences, neither in wt nor in S100Btg mice. The quantification of Aβ_42_ levels (ELISA) and ThT fluorescence in the hippocampus did not conform with each other. The comparison of one-year-old wt and S100Btg mice of both sexes revealed that Aβ_42_ was significantly increased in male S100Btg mice, while ThT fluorescence was significantly decreased in female S100Btg mice.

In the frontal cortex, ThT quantification also revealed findings opposite to the Aβ_42_ levels measured by ELISA, which measures the soluble peptide and not aggregation products. In S100Btg mice, ThT fluorescence was significantly decreased at one year of age, as compared with wt mice, and female S100Btg mice demonstrated significantly lower levels than male ones. The most likely explanation for these discrepant findings can be attributed to the fact the ELISA utilized a monoclonal antibody, recognizing the sequence 17-24 of Aβ_42_ (preferably) and Aβ_40_, both in an oligomeric and fibrillary structure. Since brain samples were homogenized in PBS, most likely, only non-fibrillary Aβ_42_ was prevalent.

In absence of an AβPP mutation in the S100Btg mouse strain, we acknowledge that a positive ThT assay does not necessarily mean that fibrillar amyloid assemblies characteristic of AD are present [49]. In fact, ThT binding is not specific for amyloid fibrillar structures of Aβ peptides, and many endogenous and exogenous factors interfere with the shift of ThT fluorescence [50,51]. Despite these methodological issues, ThT is still the most widely used “gold standard” for staining and identifying amyloid fibrils either in vivo or in vitro. In other non-AβPP mutation mouse models such as the senescence-accelerated prone mouse [52], non-transgenic BALB/c mice following intranasal infection with Chlamydia pneumonia [53], and in a high-fat diet model of sporadic AD [54] granular Aβ deposits similar to our findings (Figure 6) were demonstrated at an age of six to 18 months. The 3xTg-AD mouse strain develops at an age of four months intraneuronal Aβ pathology and extra-neuronal deposits, which become statistically different from wt mice at 6 months of age, concurrent with increased detection of insoluble Aβ_42_ [55]. Once we observed Aβ_42_ peptides, although at a lower level, in the deposits, we ventured to designate them as plaque-like structures. On the other hand, the depiction of similar aggregations, although at half the intensity in three-month-old mice in our series, challenges this interpretation.

There are limitations to this study. First, the attribution of the aggregations depicted in Aβ_42_ and ThT immunostaining as amyloid-β deposits requires additional confirmation by other methods. Second, histopathological aspects such as reactive astrogliosis and neuroinflammation have to be addressed, along with other markers that characterize AD (e.g., tau phosphorylation) and putative glial changes (e.g., apoliprotein E). Third, to better understand the timeline of aging, juvenile and 15- to 18-month-old senile mice have to be investigated. Lastly, metabolic and behavioral studies are crucial to identify involved signaling pathways and their impact on cognitive function.

## 4. Conclusions

In summary, our data reinforce the importance of this S100B transgenic mouse model for studying the effect of long-term exposure to S100B to elucidate its specific role in neurodegenerative and/or neuroadaptive processes. Since these S100Btg mice lack increased S100B levels in the adipose tissue, the model is distinguished for exploring central nervous system diseases, where CSF and serum measurements are relevant. For the first time, we reported in S100Btg mice besides increased S100B content in different brain regions, increased S100B_serum_, and S100B_CSF_ concentration. The 10-fold increase in these levels directly correlates to the number of inserted copies of the murine S100B gene and makes this model superior to other S100Btg strains, with insertion of around 70 copies of the human gene without a clear correlation to tissue levels. We demonstrated that S100Btg mice exhibit high levels of Aβ_42 CSF_ and soluble Aβ_42_ in brain tissue at one year of age with sex- and region-specific preference, i.e., in the frontal cortex in female S100Btg mice and the hippocampus in male S100Btg mice. Whether the decreased relative ThT fluorescence in the frontal cortex of one-year-old S100Btg mice of both sexes may result from neuroprotective S100B properties at nanomolar levels, thereby inducing attenuated Aβ_42_ aggregation, has to be clarified in the future. Our data emphasize the importance of this S100B transgenic model in studying neuropathological changes, particularly in Down syndrome and Alzheimer’s disease.

## 5. Materials and Methods

All animal experiments were approved by the Institutional Animal Care and Use Committee (No. TS-6/14) and following the National Research Council’s guide for the care and use of laboratory animals. Mice were kept in a controlled environment (12:12 h light/dark cycle, 22 ± 1 °C with 60% humidity). Pellet food and tap water were available ad libitum. Male and female S100Btg and wild-type (wt) mice of three months and one year of age were used in this study.

### 5.1. Polymerase Chain Reaction for Mice Genotyping

S100Btg cryo-preserved embryos were purchased from Jackson Laboratories, Charles River, Schulzfeld, Germany (C57BL/6J-Tg(S100b)5.12Rhr/J; stock no 002260) containing 12 copies of the mouse S100B gene [13]. The strain was recovered in the bio-technical laboratory of the University of Erlangen-Nürnberg and maintained by crossing carriers of Tg(S100b) 5.12Rhr to C57BL/6N mice because the transgene is homozygous lethal. Offspring were characterized by polymerase chain reaction (PCR)-based genotyping for S100B constructs. We strictly used littermates obtained from this cross-breeding strategy for all analyses. Thus, all mice used in the present study are comparable in terms of genetic background.

**Figure 7:**
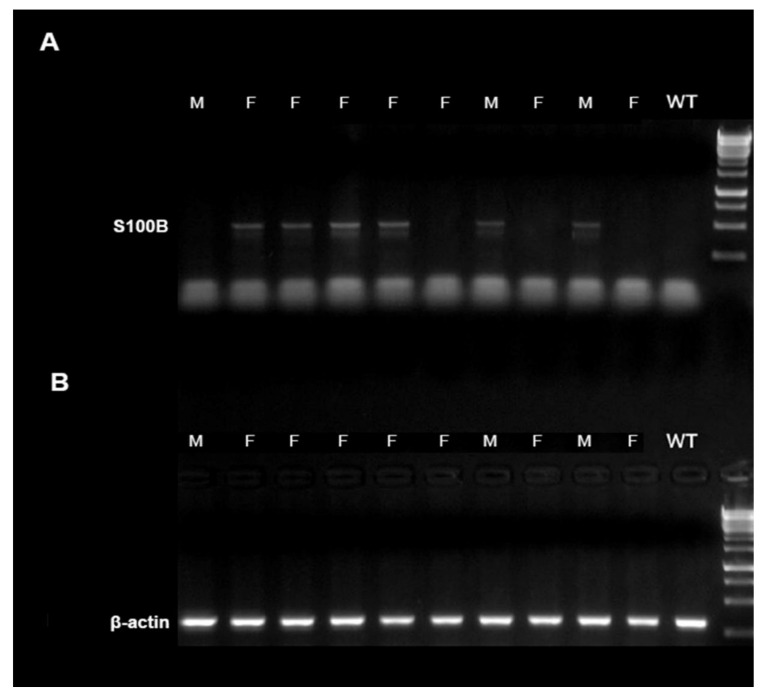
Characterization of S100Btg mice by genotyping. Genotyping of mice with the S100B gene was performed using standard polymerase chain reaction (PCR) of tail biopsies. Illustrative image of electrophoresis, in (**A**) S100B and (**B**) β-actin as control. F = female; M = male; WT = wild type.

Deoxyribonucleic acid (DNA) was isolated from the mice’s tails using HotShot–Tail–Lyse. A total of 50 µL alkaline lysis solution was added, and the samples were boiled at 95 °C for 1 h and shaken at 1200 rpm. After the samples have cooled, 50µL neutralization buffer was added. DNA could directly be used by PCR assay. The DNA for the genes encoding S100B and β-actin was performed using the primer pairs described below and Power SYBR® Green PCR Master Mix (Thermo Fisher, Waltham, MA, US) for electrophoresis analysis. Target: S100B—Sense/anti-sense: oIMR7408—F, 5′-CGAAGTTGAGATTCACAGACG-3′/oIMR7409—R, 5′-ATCATGACTGGGAAGGTTCC-3′—Product size (pb): 500. Target levels were normalized to β-actin levels. Figure 7 demonstrates an example of S100B genotyping.

### 5.2. Enzyme-Linked Immunosorbent Assay for S100B Quantification

Measurement of S100B content was performed using a commercial mouse enzyme-linked immunosorbent assay (ELISA) (LSBio- Catalog number: LS-F5980), according to the manufacturer’s instructions. This assay is based on the sandwich ELISA principle. Each well of the plate was pre-coated with a target-specific capture antibody. Standards or samples were added to the wells and the target antigen binds to the capture antibody. A biotin-conjugated detection antibody was then added to bind to the captured antigen. An avidin-horseradish peroxidase conjugate was then added to bind to the biotin. A tetramethylbenzidine substrate was then added to react with the horseradish peroxidase enzyme, resulting in color development. A sulfuric acid stop solution was added to terminate the color development reaction and the optical density of the well was measured at a wavelength of 450 nm. Protein content was measured by Bicinchoninic Acid (BCA) Protein Assay Kit (Thermo Scientific, Waltham, MA, USA), according to the manufacturer’s instructions.

### 5.3. Enzyme-Linked Immunosorbent Assay for Amyloid β_42_ Quantification

Measurement of soluble Aβ_42_ content was performed using a commercial mouse ELISA (Invitrogen- Catalog number: KMB3441), combined with colorimetric detection. Tissue and CSF were prepared according to the manufacturer’s instructions. The wells were coated with a monoclonal antibody that recognizes the N-terminus of mouse Aβ_42_. Samples and standards were added at the appropriate dilutions to the wells. The plate was incubated for 2 h at room temperature for the binding of the Aβ antigen to the capture antibody. After washing, a solution containing a rabbit antibody specific for the C-terminus of the Aβ_42_ sequence was added to the wells for 1 h at room temperature. Horseradish peroxidase-labeled anti-rabbit antibody was then added to each well for 30 min at room temperature. After washing, a solution containing the stabilized chromogen was added to each well and incubated for 20–30 min at room temperature in the dark. The reaction was stopped by a stop solution. Finally, the absorbance was determined at 450 nm. Protein content was measured by Bicinchoninic Acid (BCA) Protein Assay Kit (Thermo Scientific), according to the manufacturer’s instructions.

### 5.4. Histological Assessment

Animals were deeply anesthetized with 4% halothane in N_2_O/O_2_ (70/30%) and decapitated. The brains were then removed, fixed in 4% paraformaldehyde (Electron Microscopy Sciences, Ft. Washington, PA, USA) in 0.1 M phosphate buffer (pH 7.25) for 4 h at room temperature, blocked for coronal sections, and paraffin-embedded for microtome sectioning. A series of coronal sections (5 µm) was taken from the dorsal and rostral blade of the dentate gyrus (Bregma—1.6 mm) to the habenular commissure (Bregma—4.8 mm) throughout the hippocampus. The sections were taken using a rotary microtome, and five adjacent sections were collected every 100 µm.

Tissue was deparaffinized in xylene, rehydrated in decreasing concentrations of ethanol (100% twice, followed by once each of 95% and 70%), and rinsed with PBS triton (0.1%) for 5 min. A hydrophobic barrier pen was used to draw a waterproof barrier around the mounted sections. For the analysis, we selected coronal brain slices at dorsal hippocampal level (1.93 mm from bregma), and correspondent brain cortices. 

### 5.5. Amyloid-β Immunofluorescence and Thioflavin T Fluorescence

For amyloid-β immunostaining, sections were subjected to microwave pretreatment in 10 mM citric acid buffer, followed by hydrochloric acid at 37 °C for five minutes. This was followed by incubation with Triton X 0.4%, BSA protein 2%, and primary antibody (amyloid-β 1:500—Biolegend Clone 12F4) [24] in PBS for 48 h at 4 °C. This primary antibody was chosen according to literature and not tested for specificity [24,56,57]. Sections were rinsed 10 times with PBS plus 0.4% Triton (PBS-T) and incubated with the secondary antibody (Alexa Fluor-568) in PBS-T for 1 h. The incubation without the primary antibody excluded any unspecific staining. After rinsing with PBS, they were coverslipped with Fluorshield plus DAPI (F6057, Sigma-Aldrich, St. Louis, MO, USA).

Thioflavin T is a cationic benzothiazole dye that shows a bright yellow-green fluorescence upon binding to β sheet structures such as amyloid-β plaques or similar aggregation deposits in tissues [58]. Sections were incubated in a solution of thioflavin T (0.5% in 0.1 N HCl) in a humidity chamber for 15 min, rinsed in deionized water (3 times for 5 min), and kept in deionized water. The slices were covered with Fluor save^®^, and images were analyzed as described below. 

### 5.6. Image Analysis

Quantitative image analysis was performed as previously reported [59]. Images were acquired as digitized tagged-image format files to retain maximum resolution using an Olympus BX51 phase contrast fluorescence microscope with an attached digital camera system (DP-70, Olympus, Tokyo, Japan), and digital images were routed into a Windows PC for quantitative analyses. Image analysis was performed with Fluoviewer 3.1 FV1000 (Olympus, Tokyo, Japan [23]) and Image J (NIH). Images of three 5μm sections were captured through each anatomic region of interest (frontal cortex, entorhinal cortex, dorsal hippocampus) based on anatomical criteria defined by Franklin and Paxinos [60], and a threshold optical density was obtained that discriminated staining from the background. Each anatomic region of interest was manually edited to eliminate artifacts. For Aβ burden analyses, data are reported as the percentage of the labeled area captured (positive pixels) divided by the full area captured (total pixels). Selection bias was controlled by analyzing each region of interest in its entirety.

### 5.7. Statistical Analysis

Image analysis and quantification of optical density were performed in triplicate by two independent investigators blinded to experimental groups. Obtained data were averaged, and are presented as means ± standard error of the mean (SEM). In vivo experiments used seven animals per group. The data were analyzed by Student’s *t*-test or Pearson correlation where appropriate. Values of *p* < 0.05 were considered significant. All analyses were performed using the Graphpad Prism software version 8 (La Jolla, CA, USA).

## Figures and Tables

**Table 1:** **Quantification of relative ThT fluorescence.** In different hippocampal pathways (hilus: dynamic regulation of pattern separation; *CA3* region: termination of entorhinal cortex layer 2, novelty detection and short-term memory; temporoammonic pathway *TAP*, monosynaptic projection from entorhinal cortex layer 3 to the CA1 region, involved in spatial memory learning) and in the frontal cortex (FC) in three-month- and one-year-old wt and S100Btg mice. n.s. non significant.

		Female		*p* Value	Male		*p* Value		
Age	Brain Region	wt Mice	S100Btg Mice	wt vs. S100Btg	wt Mice	S100Btg Mice	wt vs. S100Btg	wt Sex	tg Sex
**3 months**	**Hilus** **CA3** **TAP**	3.7 ± 0.4 3.7 ± 0.3 5.1 ± 0.4	6.5 ± 0.4 4.3 ± 0.4 6.4 ± 0.4	0.033 n.s. n.s.	5.9 ± 0.8 5.8 ± 1.0 7.5 ± 1.0	7.3 ± 0.5 4.7 ± 0.4 7.6 ± 0.6	n.s. n.s. n.s.	n.s. n.s. n.s.	n.s. n.s. n.s.
***p* value**	**region**	n.s.	n.s.		n.s.	n.s.			
**1 year**	**Hilus** **CA3** **TAP**	11.9 ± 0.7 17.9 ± 1.1 13.1 ± 0.7	11.0 ± 0.7 9.2 ± 0.7 10.0 ± 0.5	n.s. n.s n.s	4.3 ± 1.1 4.7 ± 0.6 6.5 ± 0.9	10.9 ± 0.7 9.0 ± 0.6 11.4 ± 0.6	<0.001 <0.001 <0.001	<0.001 <0.001 <0.001	n.s. n.s. n.s.
***p* value**	**region**	n.s.	n.s.		n.s.	n.s.			
***p* value**	**age**	<0.001	<0.001		n.s.	<0.001			
**3 months**	**FC**	7.0 ± 0.5	6.6±0.7	n.s.	17.6 ± 1.5	10.8 ± 0.7	<0.001	<0.001	n.s.
**1 year**	**FC**	19.2 ± 1.2	3.6±0.7	<0.001	22.8 ± 0.7	12.4 ± 1.2	<0.001	n.s.	<0.001
***p* value**	**FC age**	<0.001	n.s.		0.042	n.s.			

## Data Availability

The data presented in this study are available on request from the corresponding author.
